# Optimization of Ultrasound-Assisted Extraction of Phenolic Compounds from Black Locust (*Robiniae Pseudoacaciae*) Flowers and Comparison with Conventional Methods

**DOI:** 10.3390/antiox8080248

**Published:** 2019-07-27

**Authors:** Ivana Savic Gajic, Ivan Savic, Ivana Boskov, Stanko Žerajić, Ivana Markovic, Dragoljub Gajic

**Affiliations:** 1 Faculty of Technology, University of Nis, Bulevar oslobodjenja 124, 16000 Leskovac, Serbia; 2 Technical Faculty, University of Belgrade, Vojske Jugoslavije 12, 19210 Bor, Serbia; 3 Gajic Associates, Doktora Pantica 77, 14000 Valjevo, Serbia

**Keywords:** extraction, phenolic compounds, antioxidants, central composite design, optimization

## Abstract

The aim of this study was to optimize the ultrasound-assisted extraction of phenolic compounds from black locust (*Robiniae pseudoacaciae*) flowers using central composite design. The ethanol concentration (33–67%), extraction temperature (33–67 °C), and extraction time (17–33 min) were analyzed as the factors that impact the total phenolic content. The liquid-to-solid ratio of 10 cm^3^ g^−1^ was the same during extractions. The optimal conditions were found to be 59 °C, 60% (v/v) ethanol, and extraction time of 30 min. The total phenolic content (TPC = 3.12 g_GAE_ 100 g^−1^ dry plant material) and antioxidant activity (IC_50_ = 120.5 µg cm^−3^) of the extract obtained by ultrasound-assisted extraction were compared with those obtained by maceration (TPC = 2.54 g_GAE_ 100 g^−1^ dry plant material, IC_50_ = 150.6 µg cm^−3^) and Soxhlet extraction (TPC = 3.22 g_GAE_ 100 g^−1^ dry plant material, IC_50_ = 204.2 µg cm^−3^). The ultrasound-assisted extraction gave higher total phenolic content and better antioxidant activity for shorter extraction time so that it represents the technique of choice for the extraction of phenolic compounds. The obtained extract, as the source of antioxidants, can be applied in the pharmaceutical and food industries.

## 1. Introduction

Black locust (*Robinia pseudoacacia*) is a deciduous tree originating from the Southeastern region of North America. Today, it can be found in all regions of the world with a moderate climate [1]. In Serbia, it is located in Vojvodina where it was used for bonding the “living” sand, as well as in the plains and valleys of northern Serbia. Black locust grows up to 20 m in height and 90 cm in tree diameter. Initially, its plantation was for ornamental purposes, but there are studies that mention the importance of a mixed plantation of this species, because it is a nitrogen fixing species that promotes a growing facilitation effect of other trees [2]. It has a smooth bark, thorny branches, and a rare crochet. The flowers are a cluster with characteristically white color, pleasant and intense aroma, and sweet taste.

Black locust flowers are a source of vitamin C (40 mg 100 g^−1^), resin, tannins [3], essential oil, monoterpenes (cis-β-ocimene ~26.6%, (E)-α-bergamotene ~8.9%, pinens, β-pinens, limonene), diterpene, triterpene, terpene alcohols (linalool ~33.1%), and small amount of poisonous robinin (kaempferol 3,7-di-O-glucoside) that completely disappears after thermal treatment and drying [4,5,6,7]. The content of unsaturated fatty acids in the flowers is about seven times higher than the content that of saturated fatty acids [8]. Secundiflorol, mucronulatol, isomucronulatol, and isovestitol were identified in ethanol extracts of the whole plant [9]. The presence of flavonoid glycosides, such as flavonol 3,7-di-O-glycosides, were confirmed in the black locust flower [6]. Stefova et al. [10] reported that the quercetin content is low while the kaempferol content is high in the flower extract. Sarikurkcu et al. [8] extracted the bioactive compounds from black locust flowers by the Soxhlet extractor using ethyl acetate, acetone, and methanol, and prepared the aqueous extract by maceration. The total phenolic content (TPC) and total flavonoid content (TFC) were higher in the acetone and methanol extracts compared to the aqueous extract. In order to investigate the impact of drying methods on the antioxidant properties and TPC, the ethanol extracts were prepared from the flowers previously treated by sun drying, hot-air drying, freeze-drying, and microwave-vacuum drying [11]. The highest TPC was determined in the samples treated using the freeze-drying method.

Medical use of black locust is limited only to the flower, because the other parts, especially bark, are toxic due to the high concentration of robinin [6]. It has long been used as a folk drug for alleviating the colds and coughs, stomach cramps, rheumatic pains, migraine, fever, and skin diseases [11,12]. These flowers reduce the level of cholesterol, and have diuretic and laxative effects. Phenolic compounds from the black locust flower have antioxidant [8,11,13], antimicrobial [13,14,15], and anticancer properties [10]. The techniques used for the extraction of phenolic compounds from black locust flowers are summarized in Table 1.

Because of the relatively long extraction time, large solvent consumption, higher cost of equipment, and degradation of compounds, the reflux, maceration, and Soxhlet extractions tend to be replaced by more environment-friendly extraction techniques. Ultrasound-assisted extraction (UAE) has become a good alternative method for extracting phenolic compounds since it can offer a high reproducibility for shorter extraction times and reduced consumption of the solvent [17,18]. The UAE is commonly carried out at lower temperatures so that the thermal degradation of bioactive compounds in the extract can be prevented.

TPC is related to the antioxidants, mainly phenolics in this case, in the extracts. The extracts’ phenolic composition is conditioned by liquid–solid ratio, extraction time, extraction temperature, particle size, and any other extraction variables [19]. The eco-friendly and nontoxic organic solvents are recommended by the US Food and Drug Administration for the extraction of bioactive compounds from plant materials [20]. Water and ethanol were proved as the extraction solvents of choice to reach good yields of phenolic compounds from plant materials [21,22] due to their nontoxicity. Having that in mind, the ethanolic solutions were used to extract the phenolic compounds from black locust flowers.

The one-variable-at-a-time (OVAT) approach analyzes the impact of one factor on the defined response, while all other factors are constant. In this way, the great number of experimental runs is necessary for optimizing the observed process. The interactions between the factors cannot be analyzed using the OVAT approach. The application of OVAT enables to obtain the local optimal extraction conditions, which do not correspond to the real (global) optimal conditions. In order to overcome these problems, the response surface methodology (RSM) is commonly used. This methodology predicts the system response, analyzes the interactions between the factors, defines the relationship between the response and factors, and optimizes the extraction conditions with a limited number of experiments [23,24]. Yang et al. [25] optimized the extraction parameters (extraction time, solvent concentration, and liquid-to-solid ratio) of UAE in terms of common bean (*Phaseolus vulgaris* L.) antioxidants using the two-level factorial design. Izadiyan and Hemmateenejad [26] performed multiresponse optimization of the factors (extraction temperature, solvent concentration, and extraction time) affecting the UAE of Iranian *Ocimum basilicum* using a central composite design (CCD). Yin et al. [27] applied the CCD to optimize the factors (extraction temperature, extraction time, and powder dosage) of the UAE of natural anthocyanin from purple sweet potato for silk fabric dyeing. Živković et al. [28] determined the optimal conditions for the UAE of phenolic compounds from pomegranate peel using CCD, and investigated the relationship between extraction time, solvent concentration, liquid-to-solid ratio, and extraction temperature.

Since the use of RSM has not been reported yet for modeling the UAE of phenolic compounds from black locust flowers, the aim of this study was to generate a polynomial equation that describes the extraction process. The impact of ethanol concentration, extraction temperature, and extraction time were estimated using the CCD. The optimal extraction conditions were optimized using a numerical optimization method. The identification and quantification of phenolic compounds were carried out using high-performance liquid chromatography (HPLC). The TPC and antioxidant activity of extract obtained using UAE were compared to maceration and Soxhlet extraction. The structural changes of plant materials after using these extraction techniques were observed by scanning electron microscopy (SEM).

## 2. Materials and Methods

### 2.1. Plant Materials

Black locust flowers (*Robiniae pseudoacaciae flos*) were purchased from Dr Josif Pancic (Belgrade, Serbia). The moisture content (10.6%, w/w) of the plant material was determined by measuring the weight before and after the drying at 105 °C in a hot air oven to a constant weight. The dried flowers were ground using an electric grinder to the particle size that passed through a 0.5 mm sieve.

### 2.2. Reagents

Ethanol (96%, v/v) was purchased from Zorka Pharma (Sabac, Serbia). Folin–Ciocalteu reagent, gallic acid (97%), and quercetin were purchased from Merck (Darmstadt, Germany). Rutin trihydrate (purity 97%) was purchased from Alfa Aesar (A Johnson Matthey Company, Heysham, United Kingdom). Epigallocatechin, and ferulic acid, 2,2-diphenyl-1-picrylhydrazyl (DPPH), formic acid (HPLC grade), and methanol (HPLC grade) were purchased from Sigma Aldrich (St. Louis, MO, USA).

### 2.3. Extraction of Phenolic Compounds from Black Locust Flowers

#### 2.3.1. Ultrasound-Assisted Extraction

The extractions of phenolic compounds from black locust flowers were performed in an ultrasonic cleaning bath (Sonic, Nis, Serbia) with dimensions: 30 × 15 × 20 cm. The bath was filled with distilled water up to one third of its total volume (about 3.0 dm^3^). The operating frequency was 40 kHz, while the total power was 3 × 50 W. The powder of black locust flowers (2 g) was transferred into a flask of 100 cm^3^ and extracted with 20 cm^3^ of the ethanol solution at the defined temperature. The liquid-to-solid ratio of 10 cm^3^ g^−1^ was the same during extractions, because the evaporation of the used solvents was prevented using the reflux condenser. After extraction, the flasks were immediately cooled to room temperature using chilled water. The extracts were separated from the solid matrix by vacuum filtration and subjected to further analysis. In order to determine the concentration of extracts, 1 cm^3^ of the aliquot was dried at 105 °C in a laboratory oven.

#### 2.3.2. Maceration

The powder of black locust flowers (2.0 g) was extracted with 20 cm^3^ of 60% (v/v) ethanol at 25 °C for 24 h. The extract was further treated and analyzed as in the case of UAE.

#### 2.3.3. Soxhlet Extraction

The extraction was carried out using 5.0 g of black locust flowers powder and 500 cm^3^ of 60% (v/v) ethanol at boiling temperature for 6 h. The extract was subjected to the treatment and analysis as in the previous cases.

### 2.4. Total Phenolic Compounds

The TPC was determined according to the Folin–Ciocalteu colorimetric method [29]. Briefly, 0.1 cm^3^ of the extract was mixed with 1 cm^3^ of the Folin–Ciocalteu reagent previously diluted tenfold with distilled water and 1 cm^3^ of sodium carbonate (7%, w/v). The TPC was expressed as gram of gallic acid equivalents (GAE) per 100 g dry plant material (d.p.m.). A series of methanolic solutions of gallic acid (0.005–0.300 mg cm^−3^) was prepared by diluting the stock solution (1 mg cm^−3^) to construct the calibration curve. Instead of sodium carbonate, an equivalent amount of distilled water was added to the blank solution. Absorbance of the samples was measured at 760 nm and room temperature after incubation of 90 min in relation to distilled water on the Varian Cary 100 spectrophotometer (Mulgrave, Victoria, Australia) in the quartz cuvettes (1 × 1 cm).

### 2.5. DPPH Assay

The methanolic solution of DPPH radicals (1 cm^3^) prepared to the concentration of 3 × 10^−4^ mol dm^−3^ was added to 2.5 cm^3^ of the analyzed extracts [30]. Furthermore, the blank solution composited of 1 cm^3^ of methanol and 2.5 cm^3^ of the extracts, as well as the control solution composited of 1 cm^3^ of DPPH radicals and 2.5 cm^3^ of methanol were prepared. Absorbance was measured at 517 nm in relation to methanol after incubation for 30 min at room temperature. The inhibition of DPPH radicals (%) was calculated as follows (Equation (1)):

(1) $$I_{DPPH}\left(\%\right)=\frac{A_{c}-\left(A_{s}-A_{B}\right)}{A_{c}}\times 100$$

where, ***I_DPPH_*** is the inhibition of DPPH radicals expressed in %, ***As*** is the absorbance of the samples treated with DPPH solution, ***A_B_*** is the absorbance of the blank, and ***A_C_*** is the absorbance of control.

### 2.6. HPLC Analysis

The identification and quantification of the extract’s phenolic compounds under optimal conditions were carried out using the previously described HPLC method [31]. The separation was achieved using a Zorbax Eclipse XDB-C_18_ column (4.6 × 250 mm, 5 μm) (Agilent Technologies, Santa Clara, California, USA). Based on the standards of rutin, quercetin, gallic acid, epigallocatechin, and ferulic acid, the extracts’ phenolic compounds were identified. The phenolic contents were expressed as mg 100 g^−1^ dry plant material.

### 2.7. Morphological Analysis of Black Locust Flowers

The morphology of black locust flowers before and after the UAE, maceration, and Soxhlet extraction was analyzed using the Vega-3 LMU Scanning Electron Microscope (Tescan, Brno, Czech Republic) under high vacuum conditions. The SEM was used at an accelerating voltage at 20 kV and a magnification of 2000 × (10 µm). The plant material was mounted on a metal grid with double-sided adhesive tape, and then the bead surfaces were coated with chrome under vacuum.

### 2.8. Experimental Design

The CCD with three factors was deployed to determine the optimal extraction conditions for phenolic compounds. The ethanol concentration (%, *X*_1_), extraction temperature (°C, *X*_2_) and extraction time (min, *X*_3_) were investigated at the five levels (−1.68, −1, 0, +1, +1.68). The levels of coded and actual factors are shown in Table 2. The UAE is commonly performed at the lower temperatures (20–70 °C) compared to conventional extraction procedures [32]. This method is desirable for the extraction of thermosensitive phenolic compounds from various plant species. For this reason, the temperature was observed in the range of 33–67 °C. The experimental design included the data set that belongs to the points of factorial design (2^3^), axial points (2 × 3), and central point (4). The central point was repeated four times to determine the statistical parameters of the proposed model. Due to statistical calculations, the factors *X*_i_ were coded as *x*_i_ according to Equation (2):

(2) $$x_{i}=\frac{X_{i}-X_{0}}{\delta X}$$

where *X*_0_ is the value of *X*_i_ at the central point and δ*X* is the step change. The Design Expert 12.0.0 (Stat Ease, Minneapolis, MN, USA) software was used to obtain the analysis of variance (ANOVA), regression coefficients, and regression equation. The data of response were fitted using a second-order polynomial equation (Equation (3)):

(3) $$Y=\beta_{0}+\beta_{1}x_{1}+\beta_{2}x_{2}+\beta_{3}x_{3}+\beta_{11}x_{1}^{2}+\beta_{22}x_{2}^{2}+\beta_{33}x_{3}^{2}+\beta_{12}x_{1}x_{2}+\beta_{13}x_{1}x_{3}+\beta_{23}x_{2}x_{3}+\varepsilon$$

where *Y* is the predicted response; *β*_0_ is the intercept; *β*_1_, *β*_2_, and *β*_3_ are the linear coefficients of *x*_1_, *x*_2_, *x*_3_, respectively; *β*_11_, *β*_22_, and *β*_33_ are the squared coefficients of *x*_1_, *x*_2_, and *x*_3_, respectively; *β*_12_, *β*_13_, *β*_23_ are the coefficients of interaction between *x*_1_ and *x*_2_, *x*_1_ and *x*_3_, *x*_2_ and *x*_3_, respectively; ε is the residual.

#### 2.8.1. Statistical Analysis of the Regression Model

ANOVA with 95% confidence level was carried out to analyze the significance of the model and equation terms. The sum of squares (SS), degree of freedoms (df), mean squares (MS), *F*- and *p*-values were used as the statistical parameters. The statistical significance of the terms was analyzed based on *p*-value (Prob > *F*). The model terms are statistically significant, if the *p*-value is less than 0.0500. The coefficient of determination (*R*^2^), adjusted correlation coefficient (Adj-*R*^2^), and predicted correlation coefficient (Pred-*R*^2^) were used to express the quality of the regression model. The model’s significance was checked using an *F*-test.

#### 2.8.2. Optimization of Phenolic Compounds’ Extraction

The extraction was optimized using a numerical optimization method in order to maximize the yield of phenolic compounds. Before optimization, the weighted factor was assigned to 1. The weight is important to define the form of response desirability function. It is desirable to have the value in the range of 1–10. The higher value of the weight indicates the greater importance of the response. The importance of goal was adjusted at the default value of 3. This parameter can have a value between 1 (least important) and 5 (most important).

## 3. Results and Discussion

### 3.1. Modeling of Phenolic Compounds’ Extraction Using CCD

Modeling of the extraction of phenolic compounds from black locust flowers was carried out according to the matrix of CCD with 18 experimental runs. The combinations of different factor levels and TPC are given in Table 3. The coded factors are also presented in parenthesis. TPC in the extracts was in the range 2.33–3.15 g_GAE_ 100 g^−1^ d.p.m.

The ANOVA results at 95% confidence level are depicted in Table 4. The significance of terms in the second order polynomial equation was estimated using ANOVA. The model’s *F*-value of 34.64 was higher than the critical value of 3.39 so that the model can be considered as statistically significant. Since the lack-of-fit *F*-value of 4.3 was lower than the critical value of 9.01, the lack-of-fit can be considered as not statistically significant relative to the pure error (0.0031). There is a 12.96% chance that the lack-of-fit *F*-value this large could occur due to noise. The interaction between ethanol concentration and extraction temperature, as well as the quadratic terms of ethanol concentration and extraction temperature were not statistically significant terms. The statistically significant model *F*-value and not significant lack-of-fit *F*-value indicate the adequacy of the proposed model [33].

*R*^2^ of 0.984 implies that 98.4% of the variation in the yield of phenolic compounds could be explained by the regression model (Table 4). Pred-*R*^2^ of 0.821 was in reasonable agreement with the Adj-*R*^2^ of 0.947, while Adj-*R*^2^ was close to *R*^2^. The coefficient of variation of 1.99% (C.V. < 10%) indicates the low deviation between the experimental and predicted values of the response, and the high degree of precision and reliability. Adequate precision of 19.8 indicates an adequate signal so that this model can be used to navigate the design space. This parameter is a measure of the signal to noise ratio, and it is desirable to have the value higher than 4 [34].

The polynomial model that describes the extraction process and represents the interaction between factors and response is presented in Table 5.

The not statistically significant terms could be excluded from the second order polynomial equation in order to improve the prediction ability of the proposed model. The regression coefficients indicate that the linear effects have a positive impact on the response. The quadratic effects of ethanol concentration and extraction time, as well as the interaction between ethanol concentration and extraction temperature have a negative impact on the response. The extraction time of the linear effects had the highest impact on TPC, followed by extraction temperature and ethanol concentration.

**Figure 1:**
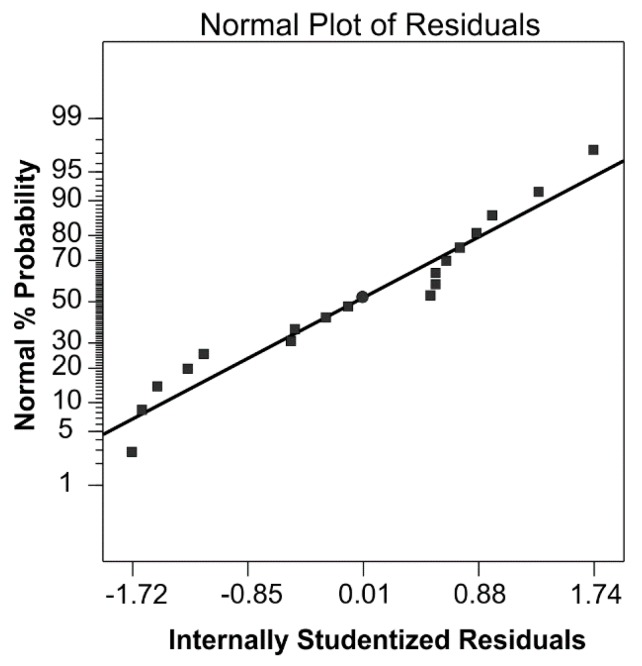
Normal probability plot of studentized residuals for the reduced polynomial model.

The adequacy of the model was also evaluated by the residuals, which represent the difference between the observed and predicted values of the response [35]. The residuals are thought of as the elements of variation unexplained by the regression model. The obtained residuals are plotted against the expected values in the normal probability plot (Figure 1). The obtained plots of the model after excluding nonstatistically significant terms indicate that the residuals are normally distributed. The slight deviation of points from the straight line in the reduced model indicates a better prediction of the regression model.

**Figure 2:**
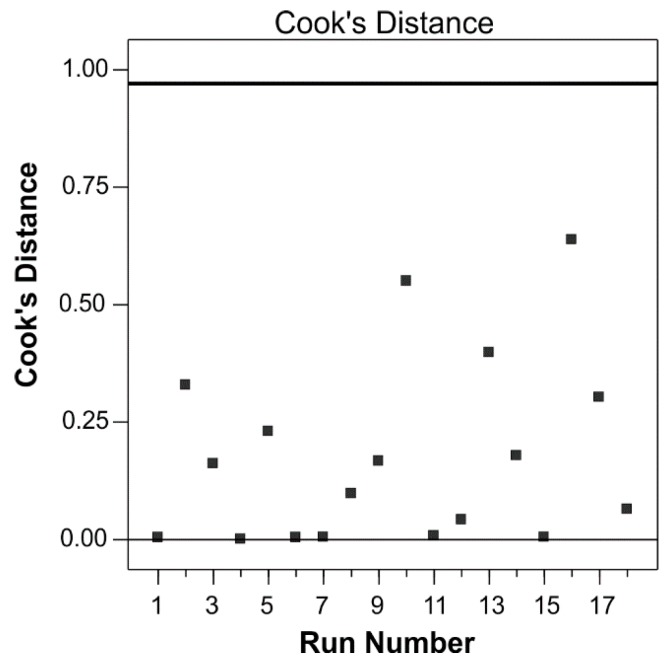
Cook’s distance for the reduced polynomial model.

Cook’s distances for the reduced polynomial model is depicted in Figure 2. Based on these values, the regression changes can be estimated when the case is deleted. Cook’s distances were less than the limit of 1.0 so that there were no outliers in the given dataset.

### 3.2. The Impacts of Factors on the Response Surface

**Figure 3:**
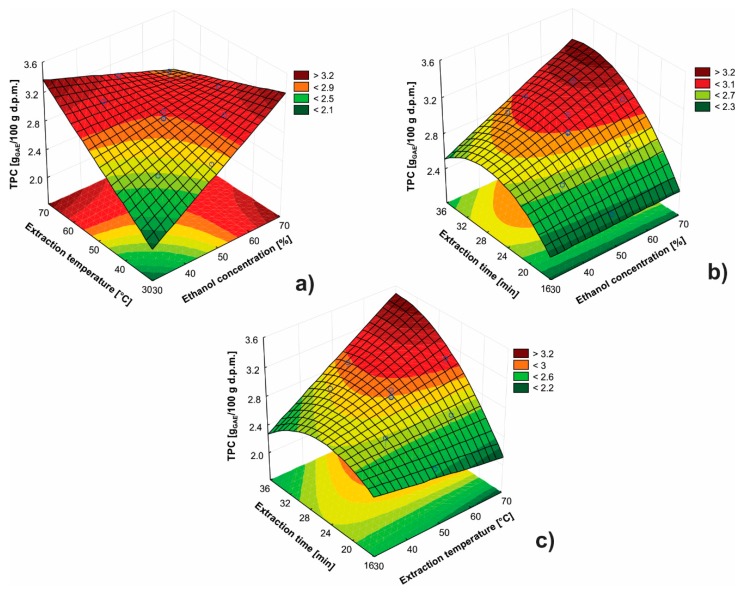
The impacts of: (**a**) ethanol concentration and extraction temperature for 25 min; (**b**) ethanol concentration and extraction time at 50 °C; and (**c**) extraction temperature and extraction time using 50% (v/v) ethanol on the TPC.

Figure 3a illustrates the interaction between ethanol concentration and extraction temperature for extraction time of 25 min. The yield of phenolic compounds increased with increasing ethanol concentration [36]. This impact on TPC is more significant for shorter extraction times. By means of analyzing the response shape, it can be concluded that there is a strong interaction between these factors. The increase of extraction temperature leads to increased TPC [36], but only using lower ethanol concentrations. The interaction between ethanol concentration and extraction time at 50 °C is depicted in Figure 3b. The impact of ethanol concentration on the response is significant at longer extraction times, while the effect of ethanol concentration at the shorter extraction times is almost negligible. The increase of extraction time is also significant at higher ethanol concentration levels and has a positive impact on the TPC [33]. Saturation in the response is achieved after extraction time of 30 min. The impacts of extraction temperature and time using 50% (v/v) ethanol are presented in Figure 3c. The extraction time has a more pronounced impact at higher extraction temperatures.

### 3.3. Optimization of the Extraction

The yield of phenolic compounds was maximized to obtain the optimal conditions for the extraction of these bioactive compounds [35]. Prior to applying the optimization method, the factor levels were ranged between −1 and +1. The optimal conditions were achieved for 60% (v/v) ethanol, 59 °C, and 30 min at the liquid-to-solid ratio of 10 cm^3^ g^−1^. The predicted TPC under these conditions was 3.17 g_GAE_ 100 g^−1^ d.p.m., while the TPC was found to be 3.12 g_GAE_ 100 g^−1^ d.p.m. Based on the good agreement between obtained and predicted TPCs, it can be concluded that the proposed model is adequate.

Sarikurkcu et al. [8] determined the TPC of 56.74 mg_GAE_ g^−1^ acetone extract, 36.42 mg_GAE_ g^−1^ methanol extract, and 27.17 mg_GAE_ g^−1^ aqueous extract of black locust flowers. Ji et al. [11] found the highest TPCs of 47.30 mg_GAE_ g^−1^ d.p.m. for the freeze-drying method, and the lowest TPC of 29.15 mg_GAE_ g^−1^ d.p.m. for sun drying method. The results of TPC obtained in this paper are in accordance with available data for different extraction techniques. Unlike previous studies, the extraction time for UAE is shorter so that the energy-efficient procedure was developed. The reduction in energy consumption was achieved by applying the ultrasound and advanced mathematical approach compared to other available procedures.

### 3.4. HPLC Analysis

The identification and quantification of phenolic compounds in the extract of black locust flowers were carried out based on the retention times and UV spectra of the standards using the reversed-phase high-performance liquid chromatographic method with ultraviolet detection (RP-HPLC–UV) The contents of rutin (56.9 mg 100 g^−1^ d.p.m., R_t_ = 32.478 min, λ_max_ = 250 nm), epigallocatechin (10.10 mg 100 g^−1^ d.p.m., R_t_ = 18.180, λ_max_ = 250 nm), ferulic acid (6.76 mg 100 g^−1^ d.p.m., R_t_ = 30.789 min, λ_max_ = 320 nm), and quercetin (2.44 mg 100 g^−1^ d.p.m., R_t_ = 50.096, λ_max_ = 250 nm) were quantified. The lowest content of identified phenolic compounds was in the case of quercetin [10]. Veitch et al. [6] identified flavonol 3,7-di-O-glycosides, flavonoid robinin, glucosyl analogue of robinin, kaempferol, and isorhamnetin in methanolic extracts obtained by maceration of black locust flowers. Truchado et al. [4] also found robinin in nectar collected from black locust flowers (Bologna, Italy) using the HPLC–MS method. In addition to these studies, there are no available data related to the chromatographic analysis of given plant material.

### 3.5. Comparison of Ultrasound-Assisted Extraction with Maceration and Soxhlet Extraction

The TPC and half maximal inhibitory concentration (IC_50_) were determined for the extracts obtained by UAE, maceration, and Soxhlet extraction to compare the efficiency of extraction techniques (Table 6). The results, which refer to the TPC, indicate that the yields of phenolic compounds are almost the same for the UAE and Soxhlet extractions. The UAE has been proven to be a more efficient and profitable extraction technique of phenolic compounds compared to the Soxhlet extraction, since the extraction time was significantly shorter [25,37]. This fact is the result of cavitation, which causes the enhancement of mass transfer of bioactive compounds through the destroyed cell walls.

The extract obtained by UAE gave better antioxidant activity than those obtained by Soxhlet extraction and maceration. The higher temperatures used in the Soxhlet extraction can probably cause the degradation of thermolabile phenolic compounds, leading to weak antioxidant activity. In the literature, there are data indicating that the methanolic extract of black locust flowers has the highest antioxidant activity (471.75 mg Trolox g^−1^ extract) compared to the extracts obtained using ethyl acetate, acetone, and water [8]. Ji et al. [11] determined that the ethanolic extracts of black locust flowers have the highest antioxidant activity previously dried by lyophilization.

The results of this study are hard to compare with available data since the extracts were obtained using different solvents and assay for the determination of antioxidant activity. The obtained black locust extracts were represented as the main source of phenolic compounds with expressed antioxidant activity that can be used in the pharmaceutical and cosmetic industries.

### 3.6. Morphological Analysis

**Figure 4:**
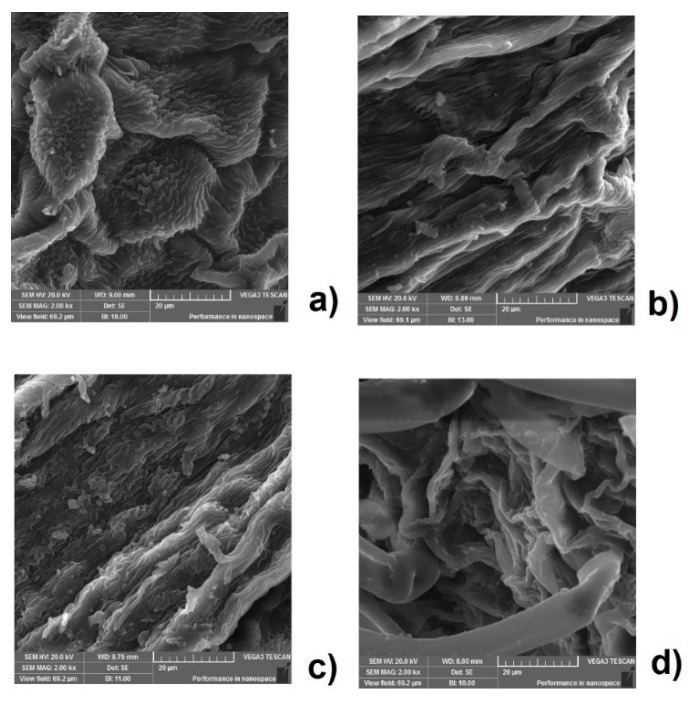
SEM (scanning electron microscopy) micrographs of black locust flowers: (**a**) without treatment; (**b**) after maceration; (**c**) after Soxhlet extraction, and (**d**) after the UAE (Ultrasound-assisted extraction).

SEM analysis was used to reveal the change in the plant material after application of different extraction techniques. The untreated flowers showed closed cells and rough surfaces (Figure 4a).

In the samples subjected to the extraction, the physical modification of cell walls with different degradation degrees can be noticed. The SEM micrographs of the sample after maceration (Figure 4b) were not considerably different from those of untreated samples, but only puny damage was observed on the external surface of the flower. In Soxhlet extraction (Figure 4c), the profuse rupture of the cellular matrix and small broken pieces were dispersed due to the treatment with high temperature. The surface of the flower was greatly destroyed after treatment with the UAE (Figure 4d). This indicates that ultrasound treatment affects the structure of the cell due to the high, localized pressures induced by cavities [38,39], which should result in the instantaneous release of the soluble compounds into the solvent. Unlike this mechanism of UAE, the maceration depends on the permeation and solubilization in order to bring the compound out of the cell [31,40].

## 4. Conclusions

The second-order polynomial model provided adequate mathematical description of the UAE of phenolic compounds from black locust flowers. ANOVA analysis confirmed that extraction time has the highest impact on the TPC, followed by the extraction temperature and ethanol concentration. The optimal extraction conditions were 60% (v/v) ethanol, 59 °C, and 30 min at the liquid-to-solid ratio of 10 cm^3^ g^−1^. Under these conditions, the TPC was found to be 3.12 g_GAE_ 100 g^−1^ d.p.m. Compared to the maceration and Soxhlet extraction, the UAE has a higher yield of phenolic compounds for shorter extraction time and better antioxidant activity. Therefore, the UAE represents an effective, reliable, and feasible technique for preparing extracts enriched with phenolic compounds, such as rutin quantified by HPLC analysis. The structural changes in the flowers obtained by the different techniques were evaluated using SEM analysis. The micrographs indicate that the different action mechanisms are characteristic for the used extraction techniques. Further studies will be directed to more detailed identification and quantification of other phenolic compounds available in the extracts obtained under optimal conditions and to investigate their biological activity.

## Figures and Tables

**Table 1:** The experimental conditions for extraction of phenolic compounds from black locust flowers previously reported in the literature.

Extraction Technique	Solvent	Temperature (°C)	Extraction Time (min)	Optimization of Factors	Ref.
Magnetic stirrer	Ethanol	Room	720	No	[11]
Reflux	Acetone	-	30	No	[10]
Maceration	Methanol	Room	-	No	[6]
Maceration	Water	100	15	No	[8]
Soxhlet	Ethyl acetate, acetone, methanol	-	300	No	[8]
Soxhlet	Ethanol	-	-	No	[14]
Microwave	Ethanol	-	2	Box–Behnken design	[16]

**Table 2:** Experimental design space for the extraction of phenolic compounds from black locust flowers.

Factors	Coded	Levels				
		−1.68	−1	0	+1	+1.68
ethanol concentration [%]	*x*_1_	33	40	50	60	67
extraction temperature [°C]	*x*_2_	33	40	50	60	67
extraction time [min]	*x*_3_	17	20	25	30	33

**Table 3:** Matrix of central composite design for three factors with total phenolic content.

Standard Order	Ru Order	*X*_1_, Ethanol Concentration [%]	*X*_2_, Extraction Temperature [°C]	*X*_3_, Extraction Time [min]	*Y*, Total Phenolic Content [g_GAE_ 100 g^−1^ d.p.m.]	
					Experimental	Predicted
11	1	50 (0)	33 (−1.68)	25 (0)	2.71	2.72
8	2	60 (+1)	60 (+1)	30 (+1)	3.15	3.19
9	3	33 (−1.68)	50 (0)	25 (0)	2.65	2.73
10	4	67 (+1.68)	50 (0)	25 (0)	3.02	3.02
5	5	40 (−1)	40 (−1)	30 (+1)	2.58	2.55
17	6	50 (0)	50 (0)	25 (0)	2.85	2.88
16	7	50 (0)	50 (0)	25 (0)	2.91	2.88
3	8	40 (−1)	60 (+1)	20 (−1)	2.71	2.69
12	9	50 (0)	67 (+1.68)	25 (0)	3.12	3.04
7	10	40 (−1)	60 (+1)	30 (+1)	3.11	3.16
18	11	50 (0)	50 (0)	25 (0)	2.92	2.88
13	12	50 (0)	50 (0)	17 (−1.68)	2.33	2.35
6	13	60 (+1)	40 (−1)	30 (+1)	3.00	3.04
2	14	60 (+1)	40 (−1)	20 (−1)	2.81	2.78
15	15	50 (0)	50 (0)	25 (0)	2.91	2.88
4	16	60 (+1)	60 (+1)	20 (−1)	2.48	2.54
14	17	50 (0)	50 (0)	33 (+1.68)	3.02	2.97
1	18	40 (−1)	40 (−1)	20 (−1)	2.48	2.46

**Table 4:** Analysis of variance (ANOVA) of the quadratic response surface model.

Source	SS	df	MS	*F*-Value	*p*-Value Prob > *F*
Model	0.9795	9	0.1088	34.64	<0.0001 *
*X*_1_	0.1023	1	0.1023	32.57	0.0005 *
*X*_2_	0.1180	1	0.1180	37.56	0.0003 *
*X*_3_	0.4652	1	0.4652	148.04	<0.0001 *
*X*_1_ *X*_2_	0.1105	1	0.1105	35.15	0.0004 *
*X*_1_ *X*_3_	0.0162	1	0.0162	5.16	0.0528 **
*X*_2_ *X*_3_	0.0761	1	0.0761	24.20	0.0012 *
*X*_1_^2^	0.0078	1	0.0078	2.50	0.1529 **
*X*_2_^2^	0.0001	1	0.0001	0.05	0.8351 **
*X*_3_^2^	0.0839	1	0.0839	26.72	0.0009 *
Residual	0.0251	8	0.0031		
Lack-of-fit	0.0221	5	0.0044	4.30	0.1296 **
Pure error	0.0031	3	0.0010		
Corrected total	1.0046	17			
Std. Dev.	0.06	*R* ^2^		0.975	
Mean	2.82	Adj-*R*^2^		0.947	
C.V. %	1.99	Pred-*R*^2^		0.821	
PRESS	0.18	Adequate precision		19.8	

* statistically significant; ** not statistically significant.

**Table 5:** Estimated regression coefficients in the polynomial equation.

Coefficient	Parameter Estimate	Standard Error
Intercept	2.898	0.028
*X*_1_	0.087	0.015
*X*_2_	0.093	0.015
*X*_3_	0.185	0.015
*X*_1_ *X*_2_	−0.118	0.020
*X*_1_ *X*_3_	0.045	0.020
*X*_2_ *X*_3_	0.098	0.020
*X*_1_^2^	−0.025	0.016
*X*_2_^2^	0.003	0.016
*X*_3_^2^	−0.082	0.016

**Table 6:** The comparison of UAE with maceration and Soxhlet extraction.

Extraction Technique	Extraction Temperature [°C]	Ethanol Concentration [%]	Extraction Time [h]	TPC [g_GAE_ 100 g^−1^ d.p.m.]	IC_50_ [µg cm^−3^]
UAE	59	60	0.5	3.12	120.5
Maceration	25	60	24	2.54	150.6
Soxhlet Extraction	90	60	6	3.22	204.2
